# Preoperative hyperglycemia is associated with elevated risk of perioperative ischemic stroke in type 2 diabetic patients undergoing non-cardiovascular surgery: A retrospective cohort study

**DOI:** 10.3389/fnagi.2022.990567

**Published:** 2022-10-20

**Authors:** Siyuan Liu, Likai Shi, Binbin Wang, Jingsheng Lou, Miao Sun, Huikai Yang, Faqiang Zhang, Min Liu, Yuxiang Song, Weidong Mi, Yulong Ma

**Affiliations:** ^1^Department of Anesthesiology, The First Medical Center of Chinese PLA General Hospital, Beijing, China; ^2^Department of Anesthesiology, Affiliated Hospital of Nantong University, Nantong, China; ^3^Department of Anesthesiology, Beijing Tongren Hospital, Capital Medical University, Beijing, China

**Keywords:** type 2 diabetes mellitus, preoperative hyperglycemia, perioperative stroke, non-cardiovascular surgery, general anesthesia

## Abstract

**Background:**

Diabetes mellitus (DM) has been reported to be associated with perioperative stroke, but the effects of preoperative hyperglycemia on the risk of perioperative stroke in diabetic patients undergoing non-cardiovascular surgery remain unclear. This study investigated the association between preoperative hyperglycemia and the risk of perioperative ischemic stroke in type 2 diabetic patients undergoing non-cardiovascular surgery.

**Methods:**

This retrospective cohort study screened 27,002 patients with type 2 DM undergoing non-cardiovascular surgery with general anesthesia between January 2008 and August 2019 at The First Medical Center of Chinese People’s Liberation Army (PLA) General Hospital. The exposure of interest was preoperative hyperglycemia, defined as a fasting plasma glucose (FPG) ≥ 7 mmol/L. The outcome of interest was a new diagnosis of perioperative ischemic stroke within 30 days after surgery. Residual confounding was minimized by controlling for observable patient and intraoperative factors. Logistic regression was conducted in the total and propensity score matched cohorts. In addition, we stratified patients into six subgroups to investigate whether the association between preoperative hyperglycemia and perioperative ischemic stroke differs in these subgroups.

**Results:**

The overall incidence of perioperative ischemic stroke was 0.53% (*n* = 144) in the current cohort. The odds of perioperative ischemic stroke were significantly increased for patients with preoperative hyperglycemia after adjusting for patient- related variables (OR: 1.95; 95% CI: 1.39–2.75; *p* < 0.001), surgery-related variables (OR: 2.1; 95% CI: 1.51–2.94; *p* < 0.001), and all confounding variables (OR: 1.78; 95% CI: 1.26–2.53; *p* < 0.001). The risk of perioperative stroke was significantly increased in patients with preoperative hyperglycemia (OR: 2.51; 95% CI: 1.66–3.9; *p* < 0.001) in the propensity score matched cohort. Preoperative hyperglycemia was associated with the outcome for all the subgroups except for patients undergoing neurosurgery.

**Conclusion:**

Preoperative hyperglycemia is associated with an elevated risk of perioperative stroke in patients with type 2 DM undergoing non-cardiovascular surgery. The effect could be eliminated for patients undergoing neurosurgery, during which specific risk factors should be considered.

## Introduction

Perioperative stroke is a severe neurological complication after surgery and is associated with considerable morbidity and mortality rates (Bateman et al., 2009; Sanders et al., 2015). The perioperative stroke incidence is approximately 0.1–1.9% in non-cardiac surgeries (Mashour et al., 2011; Woo et al., 2021) and can reach 9.7% in complicated cardiac surgeries (Bucerius et al., 2003). Despite the low incidence, perioperative stroke seriously affects the prognosis of surgical patients, and can impose extra burden on families and society.

Type 2 diabetes mellitus (DM) is characterized by persistent insulin resistance and hyperglycemia. Type 2 DM has been reported to be causally associated with ischemic stroke (Liu et al., 2018), as hyperglycemia affects arterial remodeling (Aronson and Rayfield, 2002; Beckman et al., 2002) and increases arterial stiffness (Henry et al., 2003). Hyperglycemia exerts considerable influence on diabetic microvascular pathology and is associated with an elevated risk of vascular disease (Aronson and Rayfield, 2002). Preoperative hyperglycemia is also associated with several poor clinical outcomes in a variety of surgical courses (Chuang et al., 2004; Olsen et al., 2008; Vilar-Compte et al., 2008; Chiang et al., 2021; Zhang et al., 2022) and has proven an independent predictor of perioperative stroke in patients undergoing carotid endarterectomy (Mcgirt et al., 2006).

In a multicenter, international prospective cohort study, a casual glucose level above 7.92 mmol/L before surgery was most likely to develop postoperative myocardial injury in diabetic patients undergoing non-cardiac surgery (Punthakee et al., 2018). To the best of our knowledge, a paucity of research to date has assessed the impact of preoperative hyperglycemia on perioperative stroke in non-cardiovascular surgical patients with type 2 DM. However, it is unclear whether preoperative hyperglycemia in diabetic patients is associated with an elevated risk of perioperative stroke compared to those with relatively normoglycemic status.

The goal of this retrospective cohort study is to assess the association between preoperative hyperglycemia and perioperative stroke in patients with type 2 DM undergoing non-cardiovascular surgery. We hypothesized that preoperative hyperglycemia fasting plasma glucose [(FPG) ≥ 7 mmol/L] in type 2 diabetic patients is associated with an elevated risk of perioperative stroke compared to those with normoglycemic status (FPG < 7 mmol/L).

## Materials and methods

### Study design and study population

**FIGURE 1:**
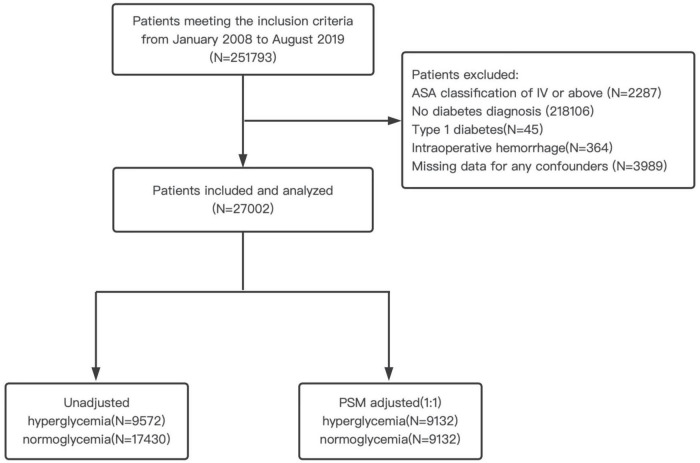
Patient flow diagram. ASA, American Society of Anesthesiologists; PSM, propensity score matching.

The Chinese People’s Liberation Army (PLA) General Hospital has a central computerized database, and there is a digital record of the demographics, diagnoses, laboratory results, and other clinical data for all inpatients. This cohort study was approved by the Medical Ethics Committee of The First Medical Center of Chinese PLA General Hospital (reference number: S2021-493-01), and the requirement for informed content was exempted. The current research adheres to the Strengthening the Reporting of Observational Studies in Epidemiology (STROBE) guidelines (Supplementary Table 1). We electronically retrieved from the database all surgical inpatients between January 2008 and August 2019. Inclusion criteria were patients aged 18 years old or older, who underwent non-cardiovascular surgery, and received general anesthesia with a surgery length of more than 1 h. A total of 251,793 adult patients met the inclusion criteria. Exclusion criteria were ASA classification of IV or above, no diabetes diagnosis, type 1 DM, intraoperative hemorrhage (transfusion of > 4 U of packed red blood cells or whole blood) and missing data for any confounders. Type 2 diabetes mellitus was identified through the discharge diagnosis of the index procedure. For patients with multiple procedures within the study period, the first surgery was used as an index procedure. In total, 27,002 participants comprised the study cohort. The patient flow diagram for this study is shown in Figure 1.

### Preoperative hyperglycemia and perioperative stroke

Preoperative glucose level was determined by the fasting plasma glucose (FPG) level at the time of preoperative evaluation, which was tested by the central laboratory. If there were multiple measurements before surgery, the value closest to the date of surgery was used, and only the results from the central laboratory were used. An FPG of ≥ 7 mmol/L is often used to diagnose diabetes, and an increase in the prevalence and incidence of diabetic retinopathy begins approximately at an FPG of 7 mmol/L (Genuth et al., 2003). Additionally, a preoperative FPG ≥ 7 mmol/L was considered to provide useful information in the perioperative setting (Duggan and Chen, 2019). Considering these points, preoperative hyperglycemia was defined as FPG ≥ 7 mmol/L. Perioperative stroke was defined as new-onset brain infarction during hospital stay. Diagnoses of stroke were confirmed by a combination of neuroimaging and clinical evidence of cerebrovascular ischemia within 30 days after surgery, identified through ICD9/ICD10 diagnosis codes (Supplementary Table 2).

### Confounding variables

The following covariates were included as potential confounders in our models: age, American Society of Anesthesiologists (ASA) classification, hypertension, coronary heart disease, heart failure, peripheral vascular disease, previous ischemic stroke, preoperative platelet, preoperative albumin, preoperative insulin, and preoperative anticoagulants were defined as patient related variables; while surgical category, surgery length, emergency surgery, intraoperative blood product usage, and intraoperative vasoactive drugs were defined as surgery-related variables.

### Statistical analysis

Continuous variables were summarized as the median and interquartile range (IQR), and categorical variables were summarized as the number and percentage of patients.

We used multivariable logistic regression for the aforementioned covariates in the analyses to determine whether preoperative hyperglycemia was independently associated with an elevated risk of perioperative ischemic stroke in diabetic patients. Ischemic stroke was modeled as the dependent variable and hyperglycemia was modeled as independent variable. Four models were built to analyze this association. Model 1 was a univariable model for crude analysis. Model 2 was a multivariable model including patient-related variables. Model 3 was a multivariable model including surgery-related variables, and model 4 included all variables. We also used the propensity score matching (PSM) method to further validate the association between preoperative hyperglycemia and perioperative stroke. In PSM, patients in the two groups were matched by propensity score (PS) at a 1:1 ratio with a caliper of 0.05.

To further investigate whether the association between preoperative hyperglycemia and perioperative stroke differs among selected patient subgroups, we also conducted multivariable logistic regression stratified by several key variables (age, sex, previous ischemic stroke, preoperative insulin, neurosurgery, and surgery length). Statistical analysis was performed using GraphPad Prism (version 9.0) and R (version 4.0), along with the MatchIt, rms, MASS, cobalt, and car packages. With all statistical tests being two-sided, a *p*-value of < 0.05 was considered statistically significant. Odds ratios with 95% confidence intervals (CI) were reported for all models.

## Results

### Characteristics of study cohort

A total of 251,793 adult patients met the inclusion criteria during the study period, and the study cohort consisted of 27,002 patients after application of exclusion criteria. Of these, 9,572 (35.4%) patients were defined as having preoperative hyperglycemia (FPG ≥ 7 mmol/L), 8,746 (32.4%) were aged ≥ 65 years old, 15,630 (57.9%) were males, 1,459 (5.4%) had a history of ischemic stroke, 13,822 (51.1%) were under insulin medication, 2,319 (8.6%) received neurosurgery, and 10,431 (38.6%) underwent a surgery length exceeding 3 h. Descriptive statistics comparing patients in the hyperglycemic group to those in the normoglycemic group in the total and PS matched cohorts are shown in Table 1.

**TABLE 1:** Patient characteristics in total and propensity score matched cohorts.

	Total cohort (*N* = 27,002)			PS matched cohort (1:1) (N = 18264)		
	FPG < 7 mmol/L (n = 17430)	FPG ≥ 7 mmol/L (n = 9572)	SMD	FPG < 7 mmol/L (n = 9132)	FPG ≥ 7 mmol/L (n = 9132)	SMD
**Patient related variables**						
Age (y)^†^	59 (51,67)	60 (53,67)	0.101	60 (53, 68)	60 (52, 67)	0.035
Male (%)^†^	9,720 (55.8)	5,910 (61.7)	0.122	5,585 (61.2)	5,556 (60.8)	0.007
BMI (kg/m^2^)	25.1 (22.9,27.5)	25.4 (23.2,27.7)	0.071	25.08 (23, 27.5)	25.39 (23.2, 27.7)	0.065
**ASA classification (%)^†^**						
I	1,007 (5.8)	374 (3.9)	0.139	344 (3.8)	367 (4)	0.013
II	13,880 (79.6)	7,387 (77.2)		7,115 (77.9)	7,086 (77.6)	
III	2,543 (14.6)	1,811 (18.9)		1,673 (18.3)	1,679 (18.4)	
Tobacco use (%)	2,016 (11.6)	1,293 (13.5)	0.059	1,238 (13.6)	1,179 (12.9)	0.019
Alcohol use (%)	2,476 (14.2)	1,467 (15.3)	0.032	1,470 (16.1)	1,342 (14.7)	0.039
**Hypertension (%)^†^**	**7,493 (43)**	3,834 (40.1)	0.06	3,709 (40.6)	3,714 (40.7)	0.001
CHD (%)	1,643 (9.4)	877 (9.2)	0.009	1,014 (11.1)	821 (9)	0.07
Heart failure (%)	27 (0.2)	17 (0.2)	0.006	16 (0.2)	15 (0.2)	0.003
Myocardial infarction (%)	183 (1)	137 (1.4)	0.034	124 (1.4)	128 (1.4)	0.004
Arrhythmia (%)	2,737 (15.7)	1,327 (13.9)	0.052	1,495 (16.4)	1,249 (13.7)	0.075
COPD (%)	184 (1.1)	89 (0.9)	0.013	107 (1.2)	84 (0.9)	0.025
Renal insufficiency (%)	280 (1.6)	128 (1.3)	0.022	149 (1.6)	121 (1.3)	0.025
Peripheral vascular disease (%)	951 (5.5)	536 (5.6)	0.006	529 (5.8)	517 (5.7)	0.006
Malignant tumor (%)	8,849 (50.8)	5,184 (54.2)	0.068	4,935 (54)	4,892 (53.6)	0.009
Previous ischemic stroke (%)	930 (5.3)	529 (5.5)	0.008	519 (5.7)	507 (5.6)	0.006
Preoperative Hb (g/L)^†^	133 (122,145)	136 (124,148)	0.136	135 (124, 147)	136 (124, 148)	0.017
Preoperative platelet (g/L)	212 (174,256)	212 (172,259)	0.008	209 (172, 252)	212 (172, 258)	0.052
Preoperative ALB (g/L)	41.1 (38.7,43.5)	41.1 (38.3,43.7)	0.042	41.10 (38.6, 43.4)	41.1 (38.4, 43.7)	0.013
Preoperative TBIL (μmol/L)^†^	10.6 (8,14.2)	11.1 (8.2,15.5)	0.181	11.1 (8.4, 14.9)	11 (8.2, 15.1)	0.027
Preoperative Oral Hypoglycemics (%)^†^	5,373 (30.8)	3,872 (40.5)	0.202	3,669 (40.2)	3,550 (38.9)	0.027
Preoperative Insulin (%)^†^	7,709 (44.2)	6,113 (63.9)	0.402	5,735 (62.8)	5,681 (62.2)	0.012
Preoperative anticoagulants (%)	1,112 (6.4)	703 (7.3)	0.038	654 (7.2)	678 (7.4)	0.01
**Anesthesia and surgery related variables**						
Emergency surgery (%)^†^	232 (1.3)	318 (3.3)	0.132	207 (2.3)	253 (2.8)	0.032
Neurosurgery (%)^†^	1,680 (9.6)	639 (6.7)	0.108	595 (6.5)	627 (6.9)	0.014
Surgery length (min)	154 (105,218)	160 (110,228)	0.071	155 (108, 217)	160 (110, 225)	0.055
Intraoperative hypotension (%)	10,269 (58.9)	5,676 (59.3)	0.008	5,278 (57.8)	5,416 (59.3)	0.031
Intraoperative vasoactive drugs (%)	3,900 (22.4)	2,265 (23.7)	0.031	2,137 (23.4)	2,136 (23.4)	< 0.001
Crystalloids infusion (ml/kg/min)	8.3 (6.2,11.1)	8.1 (6.1,10.9)	0.05	8.40 (6.3, 11.2)	8.12 (6, 10.8)	0.069
Colloids infusion (ml/kg/min)	2.6 (0,4)	2.6 (0.2,4)	0.021	2.65 (0, 4)	2.64 (0, 4)	0.024
Intraoperative blood product (%)	2,026 (11.6)	1,149 (12)	0.012	984 (10.8)	1,101 (12.1)	0.04

Data are shown as median (IQR) for continuous variables and number (percentage) for categorical variables. ^†^Variables matched in the propensity score matching. ALB, albumin; ASA, American Society of Anesthesiologists; BMI, body mass index; CHD, coronary heart disease; COPD, chronic obstructive pulmonary disease; ENT, ear, nose and throat; FPG, fasting plasma glucose; Hb, hemoglobin; IQR, Interquartile range; PS, propensity score; SMD, standardized mean difference; TBIL, total bilirubin.

### Association between preoperative hyperglycemia and perioperative stroke

Perioperative stroke significantly increased for patients in the hyperglycemic group compared to patients in the normoglycemic group (OR: 2.1; 95% CI: 1.51–2.92; *p* < 0.001). The odds of perioperative stroke for patients in the hyperglycemic group significantly increased in the logistic regression analysis after adjusting for patient-related variables (OR: 1.95; 95% CI: 1.39–2.75; *p* < 0.001), or surgery-related variables (OR: 2.1; 95% CI: 1.51–2.94; *p* < 0.001), or all confounding variables (OR: 1.78; 95% CI: 1.26–2.53; *p* < 0.001) (Table 2). The complete data of the univariate and multivariate models are detailed in Supplementary Table 3.

**TABLE 2:** Odds ratio for preoperative FPG ≥ 7 mmol/L for risk of stroke in the total and propensity score matched cohorts.

Analysis method	Odds ratio (95%CI)	*P*-value
**Logistic regression analysis (*n* = 27,002)**		
Model 1 (univariable model)	2.1 (1.51–2.92)	<0.001
Model 2 (patient related variables adjusted)	1.95 (1.39–2.75)	<0.001
Model 3 (anesthesia and surgery related variables adjusted)	2.1 (1.51–2.94)	<0.001
Model 4 (all confounding variables adjusted)	1.78 (1.26–2.53)	<0.001
**Propensity score analysis (*n* = 18,264)**		
PS matched model	2.51 (1.66–3.9)	<0.001

CI, confidence interval; FPG, fasting plasma glucose; PS, propensity score. Model 1 is a univariable model for crude analysis. Model 2 is a multivariable model including age, ASA classification, hypertension, coronary heart disease, heart failure, peripheral vascular disease, previous ischemic stroke, preoperative platelet, preoperative albumin, preoperative insulin and preoperative anticoagulants. Model 3 is a multivariable model including surgical category, surgery length, emergency surgery, intraoperative blood products usage and intraoperative vasoactive drugs. Model 4 includes all the confounding variables.

### Propensity score analysis

**FIGURE 2:**
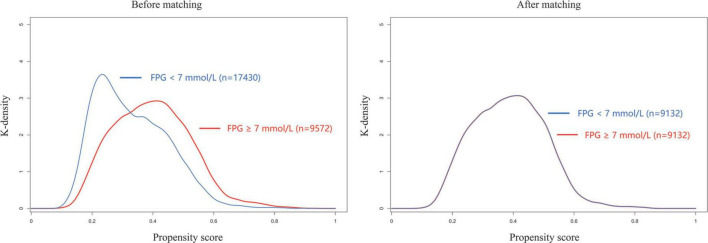
Propensity score distribution before and after matching. FPG, fasting plasma glucose.

Variables including age, sex, ASA status, hypertension, preoperative hemoglobin, preoperative total bilirubin, preoperative oral hypoglycemics, preoperative insulin, surgical category, and emergency surgery were matched in PSM. We obtained 9,132 pairs after PSM, with a standardized mean difference (SMD) of less than 0.10 for all variables (Table 1). The distribution of propensity scores in the hyperglycemic and normoglycemic groups is graphically illustrated before and after matching (Figure 2). Perioperative stroke occurred in 75 (0.82%) patients in the hyperglycemic group and 30 (0.33%) patients in the normoglycemic group in the PS matched cohort, and the risk of perioperative stroke was significantly increased in patients with preoperative hyperglycemia (OR: 2.51; 95% CI: 1.66–3.9; *p* < 0.001) (Table 2). Complete data for the PS matched cohort are detailed in Supplementary Table 4.

### Subgroup analysis

**FIGURE 3:**
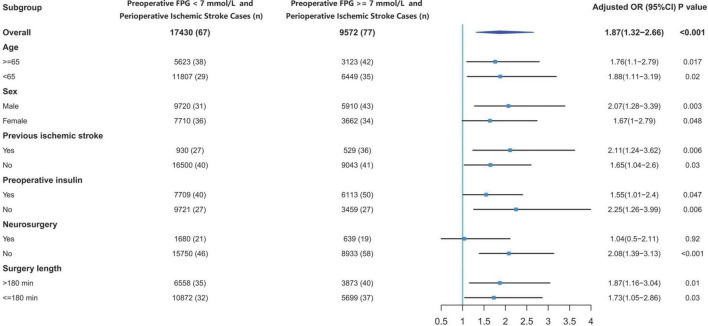
Effects of preoperative glucose on perioperative stroke risk. CI, confidence interval; FPG, fasting plasma glucose; OR, odds ratio.

The overall incidence of perioperative stroke was 0.53% (*n* = 144). The incidence was 0.8% (*n* = 77) in the hyperglycemic group and 0.38% (*n* = 67) in the normoglycemic group. We evaluated the effects of preoperative hyperglycemia on perioperative stroke in subgroups of patients stratified by age, sex, previous ischemic stroke, preoperative insulin medication, surgical category, and surgery length (Figure 3). The OR of perioperative stroke were significant in spite of age (aged ≥ 65 years old: OR: 1.76; 95% CI: 1.1–2.79; *p* = 0.017; aged < 65 years old: OR: 1.88; 95% CI: 1.11–3.19; *p* = 0.02), sex (male: OR: 2.07; 95% CI: 1.28–3.39; *p* = 0.003; female: OR: 1.67; 95% CI: 1–2.79; *p* = 0.048), previous ischemic stroke (with previous ischemic stroke: OR: 2.11; 95% CI: 1.24–3.62; *p* = 0.006; without previous ischemic stroke: OR: 1.65; 95% CI: 1.04–2.6; *p* = 0.03), preoperative insulin medication (receiving insulin medication: OR: 1.55; 95% CI: 1.01–2.4; *p* = 0.047; not receiving insulin medication: OR: 2.25; 95% CI: 1.26–3.99; *p* = 0.006) and surgery length (exceeding 3 h: OR: 1.87; 95% CI: 1.16–3.04; *p* = 0.01; within 3 h: OR: 1.73; 95% CI: 1.05–2.86; *p* = 0.03). The OR of perioperative stroke was significant for patients undergoing non-neurosurgical procedures (OR: 2.08; 95% CI: 1.39–3.13; *p* < 0.001), but not significant for patients undergoing neurosurgery (OR: 1.04; 95% CI: 0.5–2.11; *p* = 0.92).

## Discussion

Poor perioperative glycemic control increases postoperative morbidity and mortality in a variety of surgical cohorts (Chuang et al., 2004; Doenst et al., 2005; Mcgirt et al., 2006; Olsen et al., 2008; Margonis et al., 2017). In this cohort of non-cardiovascular surgical patients with type 2 DM, we found that preoperative hyperglycemia was associated with an elevated risk of perioperative stroke. Our findings will inform the role of preoperative glycemic status in optimal preoperative stroke risk assessments for diabetic patients undergoing non-cardiovascular surgery.

An overall incidence of perioperative stroke of 0.25% was recently reported in a large cohort of non-cardiac surgical patients (Woo et al., 2021). In the current cohort of 27,002 non-cardiovascular surgical patients with type 2 DM, perioperative stroke occurred in 144 (0.53%) patients. Using an FPG ≥ 7 mmol/L as primary exposure (preoperative hyperglycemia), perioperative stroke occurred in 67 (0.38%) patients with normoglycemia, and in 77 (0.8%) patients with hyperglycemia. Our results are consistent with previous researches that found that the risk of stroke ranges from approximately 0.1–1.9% in non-cardiac surgeries depending on risk factors (Mashour et al., 2011), and that diabetic patients undergoing surgical procedures have higher rates of perioperative stroke than non-diabetic patients (Vlisides and Moore, 2021). Our study demonstrated that preoperative hyperglycemia elevated the known increased risk of perioperative stroke in type 2 diabetic patients undergoing non-cardiovascular surgeries, regardless of age, sex, history of stroke, preoperative insulin medication or surgery length.

There are several possible explanations for the findings of the current cohort study. First, preoperative FPG < 7 mmol/L represents relatively tighter glycemic control, which means reduced risk factors for cardiovascular events due to improved endothelial cell function and decreased inflammatory mediators (Khatri et al., 2004; Piconi et al., 2004). Second, diabetic patients could suffer aggravated insulin resistance resulting from preoperative stress and starvation, which is described as “stress hyperglycemia,” irrespective of satisfying glucose control on ordinary days. A higher level of preoperative FPG could indicate a harsher state of insulin resistance, which is also associated with poor outcomes for surgical patients (Gillis and Carli, 2015). Finally, since poor preoperative glucose control is independently associated with postoperative hyperglycemia (Godshaw et al., 2018; Chen et al., 2019), and postoperative hyperglycemia leads to a higher rate of adverse events after surgery (Kwon et al., 2013), adverse effects of preoperative FPG ≥ 7 mmol/L could be worsened in surgical patients with type 2 DM.

We also evaluated the association between preoperative hyperglycemia and perioperative stroke across various subgroups. In our cohort of type 2 diabetic patients, the normoglycemic group showed a higher incidence of neurosurgery compared with the hyperglycemic group (9.6% vs. 6.7%). An intriguing finding in our cohort is that the association of preoperative hyperglycemia with perioperative stroke only existed in the non-neurosurgical subgroup. Presumably this is related to the fact that neurosurgical patients are exposed to very specific risks as brain tissues are very vulnerable to neurosurgical maneuvers (e.g., surgical brain injury induced by direct incisions, electrocauterization, and retraction) (Sherchan et al., 2013). It is worthwhile to note that owing to the risk of postoperative intracranial hemorrhage, anticoagulant medication was often deferred or discontinued after neurosurgeries, which also increased the incidence of thrombosis (Kreisl et al., 2008). Intraoperative mechanical insults combined with postoperative hypercoagulability predisposed neurosurgical patients to a higher risk of perioperative stroke, so that the association between preoperative hyperglycemia and perioperative stroke may become weak due to these specific risk factors. Our results indicated that patients in the hyperglycemic group were more likely to accept insulin medication than those in the normoglycemic group (63.9% vs. 44.2%). Considering that patients under insulin medication could have greater diabetes severity (resulting in more severe hyperglycemia and putting them at a higher risk of perioperative stroke), a subgroup analysis stratified by preoperative insulin medication was conducted. It turned out that preoperative hyperglycemia, regardless of whether the patients received preoperative insulin medication, was associated with an elevated risk of perioperative ischemic stroke (OR: 1.55; 95% CI: 1.01–2.4; *p* = 0.047; OR: 2.25; 95% CI: 1.26–3.99; *p* = 0.006; respectively).

The effects of preoperative hyperglycemia on the risks of postoperative complications in cardiovascular surgeries have been widely studied (Mcgirt et al., 2006; Halkos et al., 2008; Knapik et al., 2011; Kim et al., 2020; Zhang et al., 2022). Preoperative hyperglycemia was also associated with elevated postoperative infections and prolonged hospital length of stay after non-cardiac surgeries (Vilar-Compte et al., 2008; Chiang et al., 2021). The effects of preoperative glucose level on postoperative cardiovascular events during non-cardiac procedures have also received growing attention in recent years. Punthakee et al. (2018) demonstrated that preoperative casual glucose level exceeding 7.92 mmol/L was predictive of myocardial injury after non-cardiac surgery. Park et al. (2021) also reported that preoperative hyperglycemia, but not glycosylated hemoglobin levels, was associated with myocardial injury after non-cardiac surgery. As preoperative glucose level plays an important role in postoperative outcomes, satisfying preoperative glycemic control could reduce the aforementioned risks (Marchant et al., 2009; Goodenough et al., 2015; Van Den Boom et al., 2018). However, few studies have investigated the relationship between preoperative hyperglycemia and the risk of perioperative stroke after non-cardiovascular surgeries. The current study demonstrated that preoperative FPG ≥ 7 mmol/L increased the risk of perioperative stroke in non-cardiovascular surgical patients with type 2 DM.

Hyperglycemia is considered an independent predictor of adverse events after surgery, however, it is also a modifiable factor. As preoperative glucose control is a foundation of preoperative preparation for patients with DM, concerning our outcomes, preoperative FPG level is particularly important for non-cardiovascular surgical patients with type 2 DM, considering the risk of perioperative stroke. This study demonstrated that diabetic patients with preoperative hyperglycemia were more likely to suffer from perioperative stroke relative to those with normoglycemic status. Preoperative FPG ≥ 7 mmol/L could provide additional value when assessing the risk of perioperative stroke patients with type 2 DM undergoing non-cardiovascular surgeries.

There are some limitations to our findings that must be considered. In the current cohort, all the stroke cases were diagnosed postoperatively, meaning we were unable to discriminate intraoperative stroke from postoperative stroke, which are very different entities. Eleven (7.6%, data not shown) stroke cases were diagnosed 1 week after surgery, and it is difficult to identify the long-term impact of preoperative hyperglycemia on perioperative stroke risk. Nevertheless, we found a strong association between preoperative hyperglycemia and perioperative stroke. Since glycosylated hemoglobin was not a routine measurement, only 3,700 (13.7%, data not shown) subjects in the cohort had glycosylated hemoglobin results. We were unable to analyze the effect of glycosylated hemoglobin due to the significant missing data. In this respect, we highlighted the effect of short-term rather than long-term glycemic status. We conducted correlation analysis of FPG and glycosylated hemoglobin for the 3,700 participants, and we found a relatively strong correlation between preoperative FPG and glycosylated hemoglobin (*r* = 0.402, *p* < 0.001; Supplementary Figure 1). Further studies are needed to verify the effects of preoperative glycosylated hemoglobin on the risk of perioperative stroke. In addition, this is a cohort from a single institution, and a larger, multicenter cohort study is required to validate our conclusions. Our findings highlight the need for further studies to establish the ideal level of glycemic control for diabetic patients before non-cardiovascular surgeries, and for randomized clinical trials to determine whether improving glycemic control reduces the risk of perioperative stroke for diabetic patients undergoing non-cardiovascular surgeries.

## Conclusion

Preoperative hyperglycemia is associated with an elevated risk of perioperative stroke in type 2 diabetic patients undergoing non-cardiovascular surgery. The effect could be eliminated for patients undergoing neurosurgery, during which specific risk factors should be taken into consideration. As preoperative fasting plasma glucose ≥ 7 mmol/L predisposes type 2 diabetic patients to perioperative stroke, clinicians should take note of fasting plasma glucose level during preoperative evaluation.

## Data availability statement

The original contributions presented in this study are included in the article/Supplementary material, further inquiries can be directed to the corresponding author/s.

## Ethics statement

The studies involving human participants were reviewed and approved by the Medical Ethics Committee of The First Medical Center of Chinese PLA General Hospital. The ethics committee waived the requirement of written informed consent for participation.

## Author contributions

SL wrote the manuscript with contributions from all authors. WM, YM, and SL designed the study. LS, MS, FZ, HY, JL, ML, and YS were responsible for data extraction and acquisition. YM, SL, and BW designed and conducted the statistical analyses. All authors critically reviewed the report and approved the final version.
