# Supplementary material for: Preoperative hyperglycemia is associated with elevated risk of perioperative ischemic stroke in type 2 diabetic patients undergoing non-cardiovascular surgery: A retrospective cohort study
Source: Front Aging Neurosci. 2022 Oct 20;14:990567. doi: 10.3389/fnagi.2022.990567 (PMC9631439; doi:10.3389/fnagi.2022.990567)
Supplement: Supplementary file 1 [file Data_Sheet_1.docx]

Supplementary Table 1. STROBE Statement—Checklist of Items that Should Be Included in Reports of Cohort Studies

|  | Item No | Recommendation | Page No |
| --- | --- | --- | --- |
| **Title and abstract** | 1 | (*a*) Indicate the study’s design with a commonly used term in the title or the abstract | 1, 2 |
|  |  | (*b*) Provide in the abstract an informative and balanced summary of what was done and what was found | 2 |
| Introduction | | |  |
| Background/rationale | 2 | Explain the scientific background and rationale for the investigation being reported | 2,3 |
| Objectives | 3 | State specific objectives, including any prespecified hypotheses | 3 |
| Methods | | |  |
| Study design | 4 | Present key elements of study design early in the paper | 3 |
| Setting | 5 | Describe the setting, locations, and relevant dates, including periods of recruitment, exposure, follow-up, and data collection | 3,4 |
| Participants | 6 | (*a*) Give the eligibility criteria, and the sources and methods of selection of participants. Describe methods of follow-up | 3,4 |
|  |  | (*b*) For matched studies, give matching criteria and number of exposed and unexposed | 4 |
| Variables | 7 | Clearly define all outcomes, exposures, predictors, potential confounders, and effect modifiers. Give diagnostic criteria, if applicable | 3,4 |
| Data sources/ measurement | 8* | For each variable of interest, give sources of data and details of methods of assessment (measurement). Describe comparability of assessment methods if there is more than one group | 3,4 |
| Bias | 9 | Describe any efforts to address potential sources of bias | 4 |
| Study size | 10 | Explain how the study size was arrived at | 5 |
| Quantitative variables | 11 | Explain how quantitative variables were handled in the analyses. If applicable, describe which groupings were chosen and why | 4 |
| Statistical methods | 12 | (*a*) Describe all statistical methods, including those used to control for confounding | 4 |
|  |  | (*b*) Describe any methods used to examine subgroups and interactions | 4 |
|  |  | (*c*) Explain how missing data were addressed | Not applicable |
|  |  | (*d*) If applicable, explain how loss to follow-up was addressed | Not applicable |
|  |  | (*e*) Describe any sensitivity analyses | 4 |
| Results | | |  |
| Participants | 13* | (a) Report numbers of individuals at each stage of study—eg numbers potentially eligible, examined for eligibility, confirmed eligible, included in the study, completing follow-up, and analysed | 5 |
|  |  | (b) Give reasons for non-participation at each stage | Not applicable |
|  |  | (c) Consider use of a flow diagram | Figure 1 |
| Descriptive data | 14* | (a) Give characteristics of study participants (eg demographic, clinical, social) and information on exposures and potential confounders | Table 1 |
|  |  | (b) Indicate number of participants with missing data for each variable of interest | Not applicable |
|  |  | (c) Summarise follow-up time (eg, average and total amount) | Not applicable |
| Outcome data | 15* | Report numbers of outcome events or summary measures over time | 5 |
| Main results | 16 | (*a*) Give unadjusted estimates and, if applicable, confounder-adjusted estimates and their precision (eg, 95% confidence interval). Make clear which confounders were adjusted for and why they were included | Table 2, Supplementary Table 3, Supplementary Table 4 |
|  |  | (*b*) Report category boundaries when continuous variables were categorized | 5 |
|  |  | (*c*) If relevant, consider translating estimates of relative risk into absolute risk for a meaningful time period | Not applicable |
| Other analyses | 17 | Report other analyses done—eg analyses of subgroups and interactions, and sensitivity analyses | 5,6 |
| Discussion | | |  |
| Key results | 18 | Summarise key results with reference to study objectives | 6-8 |
| Limitations | 19 | Discuss limitations of the study, taking into account sources of potential bias or imprecision. Discuss both direction and magnitude of any potential bias | 8 |
| Interpretation | 20 | Give a cautious overall interpretation of results considering objectives, limitations, multiplicity of analyses, results from similar studies, and other relevant evidence | 6-8 |
| Generalisability | 21 | Discuss the generalisability (external validity) of the study results | 8 |
| Other information | | |  |
| Funding | 22 | Give the source of funding and the role of the funders for the present study and, if applicable, for the original study on which the present article is based | 9 |

Supplementary Table 2. ICD-9/10 Diagnosis Codes for Ischemic Stroke

| Ischemic stroke | ICD-9/ICD-10 | 433.X1/I63.X | Occlusion and stenosis of precerebral arteries with cerebral infarction |
| --- | --- | --- | --- |
|  | ICD-9 | 434.X1 | Occlusion of cerebral arteries with cerebral infarction |
|  | ICD-9/ICD-10 | 437.1/I67.81,  I67.89 | Other generalized ischemic cerebrovascular disease |
|  | ICD-9/ICD-10 | 437.9/I67.9 | Unspecified cerebrovascular disease |

Supplementary Table 3. Univariate and Multivariate Logistic Regression Analysis for Perioperative Stroke in Model 4

| Variables | Univariate analysis |  | Multivariate analysis |  |
| --- | --- | --- | --- | --- |
|  | OR (95% CI) | P value | OR (95% CI) | P value |
| Preoperative hyperglycemia (Yes vs No) | 2.102 (1.514–2.924) | < 0.001 | 1.785 (1.264-2.526) | 0.001 |
| Age | 1.052 (1.035–1.069) | <0.001 | 1.041 (1.022-1.061) | < 0.001 |
| Sex (male vs female) | 1.302 (0.937–1.807) | 0.114 |  |  |
| BMI | 1.004 (0.959–1.049) | 0.869 |  |  |
| ASA classification  Class I | reference |  |  |  |
| Class II | 1.93 (0.724–7.867) | 0.263 | 1.16(0.421-4.805) | 0.804 |
| Class III | 5.552 (2.042–22.833) | 0.004 | 1.45(0.5-6.19) | 0.547 |
| Tobacco use (Yes vs No) | 0.591(0.301-1.043) | 0.094 |  |  |
| Alcohol use (Yes vs No) | 0.579(0.311-0.985) | 0.061 |  |  |
| Hypertension (Yes vs No) | 2.249 (1.611-3.167) | < 0.001 | 1.222 (0.849-1.771) | 0.284 |
| CHD (Yes vs No) | 2.255 (1.452-3.378) | < 0.001 | 1.309 (0.814-2.041) | 0.249 |
| Heart failure (Yes vs No) | 8.993 (1.457-29.568) | 0.003 | 2.633 (0.38-10.842) | 0.237 |
| Myocardial infarction (Yes vs No) | 1.781 (0.438-4.732) | 0.325 |  |  |
| Arrhythmia (Yes vs No) | 1.245 (0.796-1.874) | 0.313 |  |  |
| COPD (Yes vs No) | 0.684(0.039-3.062) | 0.705 |  |  |
| Renal insufficiency (Yes vs No) | 1.39(0.342-3.685) | 0.574 |  |  |
| Peripheral vascular disease (Yes vs No) | 5.404 (3.614-7.876) | < 0.001 | 1.832 (1.148-2.858) | 0.009 |
| Malignant tumor (Yes vs No) | 0.781 (0.561-1.084) | 0.14 |  |  |
| Previous ischemic stroke (Yes vs No) | 14.186 (10.132-19.775) | < 0.001 | 8.1 (5.576-11.719) | < 0.001 |
| Preoperative hemoglobin | 0.992 (0.983-1.001) | 0.073 |  |  |
| Preoperative platelet | 1.004(1.002-1.006) | < 0.001 | 1.004(1.002-1.006) | < 0.001 |
| Preoperative albumin | 0.947 (0.911-0.985) | 0.005 | 0.994(0.955-1.035) | 0.771 |
| Preoperative total bilirubin | 1(0.994-1.003) | 0.836 |  |  |
| Preoperative Oral Hypoglycemics (Yes vs No) | 1.119 (0.792-1.565) | 0.515 |  |  |
| Preoperative Insulin (Yes vs No) | 1.593(1.14-2.247) | 0.007 | 1.244(0.87-1.792) | 0.235 |
| Preoperative anticoagulants (Yes vs No) | 2.252 (1.36-3.534) | < 0.001 | 1.015(0.588-1.676) | 0.954 |
| Emergency surgery (Yes vs No) | 4.447 (2.321-7.743) | < 0.001 | 3.151 (1.553-5.872) | < 0.001 |
| Neurosurgery (Yes vs No) | 4.148 (2.842-5.937) | < 0.001 | 3.583(2.288-5.527) | < 0.001 |
| Surgery length | 1.002 (1.001–1.004) | < 0.001 | 1.002 (1-1.003) | 0.018 |
| Intraoperative hypotension (Yes vs No) | 1.389(0.987-1.981) | 0.064 |  |  |
| Intraoperative vasoactive drugs (Yes vs No) | 1.54 (1.072-2.179) | 0.017 | 1.11 (0.76-1.599) | 0.58 |
| Crystalloid infusion | 0.978 (0.935-1.02) | 0.31 |  |  |
| Colloid infusion | 1.038 (0.966-1.111) | 0.299 |  |  |
| Intraoperative blood products (Yes vs No) | 1.738 (1.119-2.602) | 0.01 | 1.354 (0.833-2.138) | 0.206 |

Abbreviations: ASA, American Society of Anesthesiologists; BMI, body mass index; CHD, coronary heart disease; COPD, chronic obstructive pulmonary disease; CI, confidence interval; OR, odds ratio.

Supplementary Table 4. Univariate Logistic Regression Analysis for Perioperative Stroke in the PS Matched Cohort.

| Variables | Univariate analysis |  |
| --- | --- | --- |
|  | OR (95% CI) | P value |
| Preoperative hyperglycemia (Yes vs No) | 2.512 (1.662–3.897) | < 0.001 |
| Age | 1.053 (1.033–1.074) | <0.001 |
| Sex (male vs female) | 1.319 (0.895-1.937) | 0.158 |
| BMI | 1.015 (0.962–1.067) | 0.585 |
| ASA classification  Class I | reference |  |
| Class II | 3.113 (0.687-55.048) | 0.26 |
| Class III | 9.009 (1.96-159.881) | 0.03 |
| Tobacco use (Yes vs No) | 0.467(0.196-0.934) | 0.052 |
| Alcohol use (Yes vs No) | 0.642(0.324-1.146) | 0.165 |
| Hypertension (Yes vs No) | 2.291 (1.553-3.418) | < 0.001 |
| CHD (Yes vs No) | 2.253 (1.358-3.57) | < 0.001 |
| Heart failure (Yes vs No) | 12.139 (1.946-41) | < 0.001 |
| Myocardial infarction (Yes vs No) | 1.391 (0.229-4.417) | 0.645 |
| Arrhythmia (Yes vs No) | 1.417 (0.855-2.244) | 0.154 |
| COPD (Yes vs No) | 0.909(0.051-4.104) | 0.925 |
| Renal insufficiency (Yes vs No) | 1.971(0.483-5.276) | 0.249 |
| Peripheral vascular disease (Yes vs No) | 5.822 (3.678-8.94) | < 0.001 |
| Malignant tumor (Yes vs No) | 0.668 (0.452-0.981) | 0.041 |
| Previous ischemic stroke (Yes vs No) | 15.388 (10.403-22.69) | < 0.001 |
| Preoperative hemoglobin | 0.996 (0.986-1.007) | 0.453 |
| Preoperative platelet | 1.004(1.002-1.006) | < 0.001 |
| Preoperative albumin | 0.946 (0.906-0.99) | 0.015 |
| Preoperative total bilirubin | 0.999(0.991-1.003) | 0.63 |
| Preoperative Oral Hypoglycemics (Yes vs No) | 0.98 (0.657-1.446) | 0.92 |
| Preoperative Insulin (Yes vs No) | 1.435(0.952-2.214) | 0.092 |
| Preoperative anticoagulants (Yes vs No) | 2.301 (1.299-3.817) | 0.002 |
| Emergency surgery (Yes vs No) | 5.101 (2.637-9.003) | < 0.001 |
| Neurosurgery (Yes vs No) | 4.914 (3.106-7.54) | < 0.001 |
| Surgery length | 1.002 (1–1.004) | 0.009 |
| Intraoperative hypotension (Yes vs No) | 1.619(1.078-2.487) | 0.023 |
| Intraoperative vasoactive drugs (Yes vs No) | 1.714 (1.133-2.551) | 0.009 |
| Crystalloid infusion | 0.985 (0.935-1.033) | 0.559 |
| Colloid infusion | 1.031 (0.947-1.116) | 0.47 |
| Intraoperative blood products (Yes vs No) | 1.834 (1.094-2.927) | 0.015 |

Abbreviations: ASA, American Society of Anesthesiologists; BMI, body mass index; CHD, coronary heart disease; COPD, chronic obstructive pulmonary disease; CI, confidence interval; OR, odds ratio.
