# Supplementary figures and images for: Preoperative hyperglycemia is associated with elevated risk of perioperative ischemic stroke in type 2 diabetic patients undergoing non-cardiovascular surgery: A retrospective cohort study
Source: Front Aging Neurosci. 2022 Oct 20;14:990567. doi: 10.3389/fnagi.2022.990567 (PMC9631439; doi:10.3389/fnagi.2022.990567)

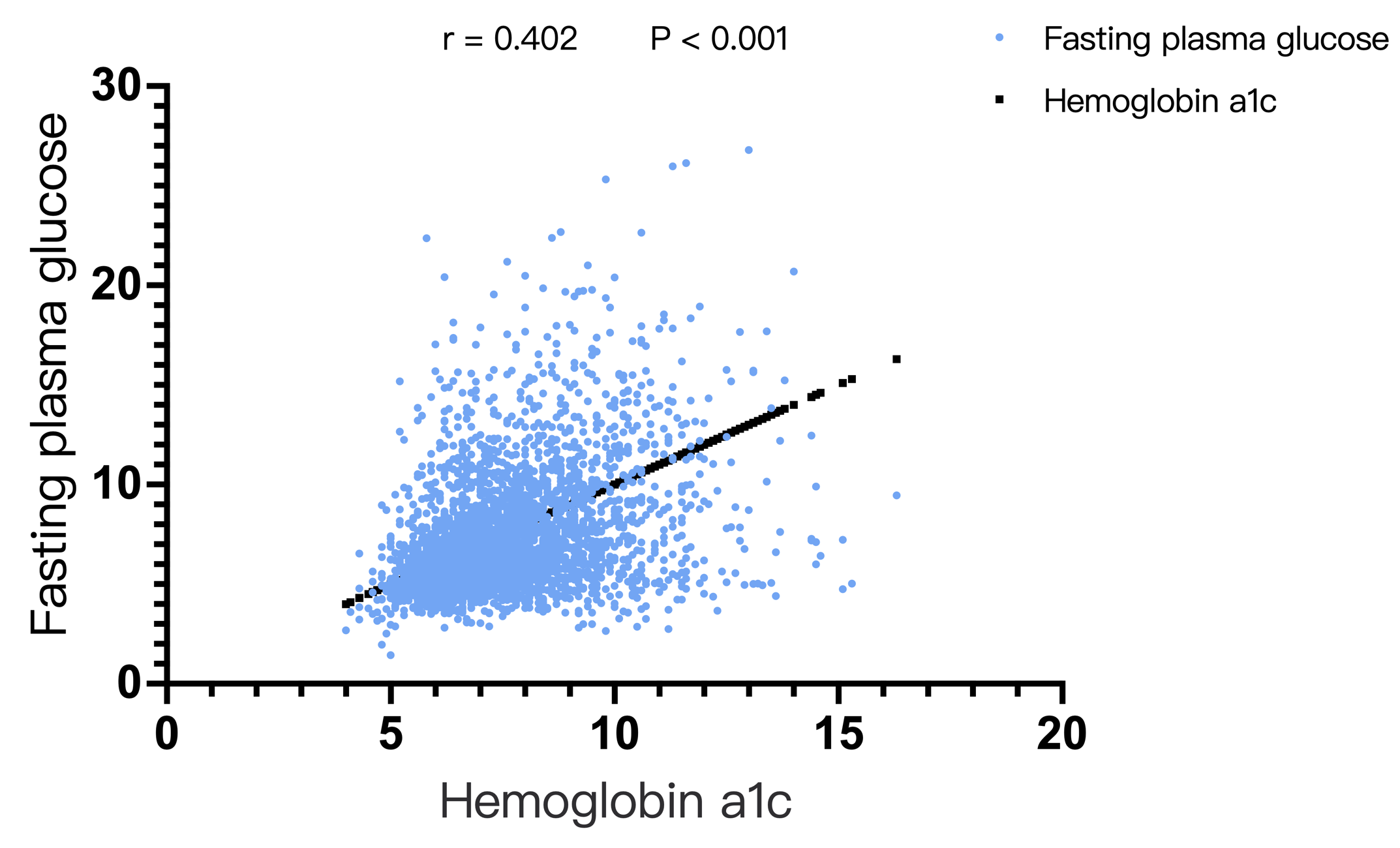


Supplementary Figure 1. Correlation Analysis of Fasting Plasma Glucose and Hemoglobin a1c, n = 3700.

Supplement: Supplementary file 2 [file Data_Sheet_2.docx]
